# Oxytocin facilitates social approach behavior in women

**DOI:** 10.3389/fnbeh.2014.00191

**Published:** 2014-05-27

**Authors:** Katrin Preckel, Dirk Scheele, Keith M. Kendrick, Wolfgang Maier, René Hurlemann

**Affiliations:** ^1^Department of Psychiatry, University of BonnBonn, Germany; ^2^Department of Medical Psychology, University of BonnBonn, Germany; ^3^Key Laboratory for Neuroinformation, School of Life Science and Technology, University of Electronic Science and Technology of ChinaChengdu, China; ^4^German Center for Neurodegenerative Diseases (DZNE)Bonn, Germany

**Keywords:** approach, avoidance, female, sexual dimorphic, oxytocin, social distance

## Abstract

In challenging environments including both numerous threats and scarce resources, the survival of an organism depends on its ability to quickly escape from dangers and to seize opportunities to gain rewards. The phylogenetically ancient neurohormonal oxytocin (OXT) system has been shown to influence both approach and avoidance (AA) behavior in men, but evidence for comparable effects in women is still lacking. We thus conducted a series of pharmacological behavioral experiments in a randomized double-blind study involving 76 healthy heterosexual women treated with either OXT (24 IU) or placebo intranasally. In Experiment 1, we tested how OXT influenced the social distance subjects maintained between themselves and either a female or male experimenter. In Experiment 2, we applied a reaction time based AA task. In Experiment 3 we investigated effects on peri-personal space by measuring the lateral attentional bias in a line bisection task. We found that OXT specifically decreased the distance maintained between subjects and the male but not the female experimenter and also accelerated approach toward pleasant social stimuli in the AA task. However, OXT did not influence the size of peri-personal space, suggesting that it does not alter perception of personal space *per se*, but rather that a social element is necessary for OXT's effects on AA behavior to become evident. Taken together, our results point to an evolutionarily adaptive mechanism by which OXT in women selectively promotes approach behavior in positive social contexts.

## Introduction

For humans as with all social species, approach behavior has a pivotal function in signaling interest in conspecifics, thereby providing a means to establish and cultivate close relationships (Baumeister and Leary, 1995). The phylogenetically ancient neuropeptide oxytocin (OXT) has been implicated in modulating both approach/avoidance (AA) behavior and pair-bonding in men (Kemp and Guastella, 2011; Scheele et al., 2012; Striepens et al., 2014). However, it is still unknown whether OXT influences these fundamental behavioral tendencies equivalently in both sexes.

Despite mounting evidence that OXT can affect a broad repertoire of sophisticated human behaviors (Kosfeld et al., 2005; Hurlemann et al., 2010; Bartz et al., 2011; Bos et al., 2012; Bethlehem et al., 2014), few studies have examined the peptide's effect on AA behavior as a probe for appetitive and aversive motivation. We have shown that intranasal OXT administration motivates men in a monogamous heterosexual relationship, but not single men, to keep a larger social distance from unfamiliar attractive women, suggesting that OXT may strengthen the existing monogamous pair-bond (Scheele et al., 2012). On the other hand, Liu et al. (2012) failed to find an effect of OXT administered to both interacting male and female subjects on the physical distance maintained between them using a microcoded video-recording approach. However, since the experimental design in their study included concomitant treatment of both male and female subjects (Liu et al., 2012) the influence that cross-partner interaction has on the readiness for social engagement (Weisman et al., 2012) may have interfered with OXT effects. Another study utilizing a reaction time based AA task observed that OXT diminished the differential response to angry and happy faces in men (Radke et al., 2013). These findings are in stark contrast with the results of another study reporting an accelerated response selectively for disgusted facial expressions (Theodoridou et al., 2013) and where the authors propose that OXT may play a role in behavioral prophylaxis. This latter interpretation resonates well with previous findings of enhanced defensive reactions after OXT administration (Striepens et al., 2012; Grillon et al., 2013). Thus, it appears OXT can promote approach behavior in men, but only under certain circumstances and with heightened caution (Striepens et al., 2012).

Sexual-dimorphic effects of OXT are not surprising, since gonadal steroids play a role in early morphogenesis of the OXT system (De Vries, 2008), influence the production of OXT (Patisaul et al., 2003), and regulate the density of OXT receptors (OXTR) and OXTR binding (Johnson et al., 1991; Young et al., 1998; Choleris et al., 2008). In female prairie voles, OXT is critical for the formation of a partner preference, while in male voles, the related neuropeptide vasopressin appears to be more important for pair bonding (Insel and Hulihan, 1995; Cushing et al., 2001; Ross et al., 2009). In humans, common OXTR gene variants interact with sex to predict harm avoidance (Stankova et al., 2012) and the behavioral and neural effect profile of intranasally administered OXT is profoundly different between male and female players of an interactive social game (Rilling et al., 2014). Furthermore, Fischer-Shofty et al. (2013) reported that OXT improves kinship recognition in women and competition recognition in men and Herzman et al. (2013) found that OXT impairs recollection judgments only in men, but not in women. Perhaps the most prominent sex-specific OXT effect is the peptide's modulatory influence on amygdalar activity. OXT suppresses amygdala responses to fear-inducing visual stimuli in men (Domes et al., 2007; Kirsch et al., 2005), but it enhances responses to threatening scenes in women (Domes et al., 2010; Lischke et al., 2012). However, sex differences are not evident in all behavioral domains (Cardoso et al., 2012) and there is also evidence for a decreased amygdala responsiveness after OXT treatment in nulliparous women (Rupp et al., 2014).

To test potential sex-specific effects of OXT on AA behavior, we submitted 76 healthy heterosexual women to a series of randomized, placebo-controlled between-subject design experiments, which we had previously carried out in males (Scheele et al., 2012). In Experiment 1 we applied a stop distance paradigm to measure the social distance maintained by subjects when encountering an unfamiliar male or female experimenter. In Experiment 2 we assessed reaction times in a computerized AA task entailing a broad range of pleasant and aversive emotional scenes. Lastly, in Experiment 3, we probed whether there needs to be a social context for OXT to unfold its effects by using a modified line bisection task which provides an estimate of the extent or “size” of the subjects' non-social peri-personal space.

## Materials and methods

### Subjects and protocols

A total of 76 healthy heterosexual female adults (mean age ± *SD* = 23.76 ± 2.60) participated in this study after giving written informed consent. In Experiment 2, 9 participants were excluded, because their reaction time was either more than two SDs above or below the mean. In Experiment 3, four left-handed subjects were excluded. The study was approved by the institutional review board of the Medical Faculty of the University of Bonn (Germany) and was performed in compliance with the latest revision of the Declaration of Helsinki. All subjects were free of current and past physical or psychiatric illness, as assessed by medical history and the Mini-International Neuropsychiatric Interview (M.I.N.I.) (Sheehan et al., 1998). Participants also completed a comprehensive neuropsychological test battery including the Social Interaction Anxiety Scale (SIAS) and Social Phobia Scale (SPS) (Heinrichs et al., 2002), the Family Conflict Resolution Scale (German abbreviation: KLSE) (Kog et al., 1987), the Positive and Negative Affect Schedule (PANAS) (Krohne et al., 1996), the State and Trait Anxiety Inventory (STAI-S and STAI-T) (Englert et al., 2011), and the Letter-Number-Sequence (BZF) (Schächtele, 2009). In total, the screening and test session lasted approximately 1.5 and 2 h, respectively. All female participants were not pregnant, not lactating and used oral contraceptives. Nineteen participants in the OXT group and 20 participants in the PLC were in a stable monogamous relationship. Estimation of which treatment was received was comparable between the OXT and PLC groups [χ^2(1)^ = 0.91; *P* = 0.34], indicating that subjects were unaware of whether they had received OXT or PLC. Participants were asked to maintain their regular sleep-wake cycle and to abstain from caffeine and alcohol intake on the day of the experiment. In addition, they were naive to prescription-strength psychoactive medication, and had not taken any over-the-counter psychoactive medication in the past 4 weeks. There were no significant pretreatment differences (Table 1). Personality traits possibly affecting the attitude toward social distance were also assessed (e.g., SIAS and SPS) (Mattick and Clarke, 1998). Using a double-blind, counter-balanced, randomized, parallel-group design, either intranasal OXT (*n* = 38) (24 IU, Syntocinon-Spray, Sigma Tau; 3 puffs per nostril, each with 4 IU OXT) or PLC (*n* = 38) (PLC, containing all ingredients except for the peptide) was given 45 min before the start of the experiments. The intranasal administration of OXT has been shown to increase not only peripheral OXT plasma levels but also central OXT concentration in the cerebrospinal fluid (Striepens et al., 2013). Furthermore, Born et al. (2002) demonstrated that the intranasal administration of the related peptide arginine vasopressin (AVP) increases lumbar cerebrospinal fluid concentrations of AVP after 10 min, and that the AVP level peaks after 80 min.

**Table 1:** **Demographics and neuropsychological performance**.

	**OXT (*N* = 38)**	**PLC (*N* = 38)**
Age (years)	23.61 (2.71)	23.74 (2.47)
Education (years)^a^	15.88 (4.36)	14.44 (5.71)
Months of being single^a^	5.62 (19.06)	13.74 (13.69)
Months of being in a relationship^a^	34.16 (28.48)	32.63 (27.67)
Social Interaction Anxiety Scale (SIAS)^a^	16.13 (11.11)	17.97 (12.12)
Social Phobia Scale (SPS)^a^	6.66 (7.19)	7.73 (8.49)
Family conflict resolution (KLSE)	31.89 (4.63)	32.5 (5.51)
Positive affect (PANAS)	32.79 (6.31)	30.29 (6.22)
Negative affect (PANAS)^a^	44.82 (4.16)	43.13 (7.45)
State anxiety (STAI)	1.76 (0.36)	1.83 (0.23)
Trait anxiety (STAI)^a^	2.14 (0.17)	2.14 (0.23)
Visual attention (D2)	13.32 (2.45)	13.09 (2.84)
Letter-number-span-test (BZT)^a^	17.76 (2.47)	17.66 (2.12)

Given are mean values (SD). There were no significant differences between the OXT and PLC group (all Ps > 0.05).

a Data derived from not normally distributed populations were compared by using non-parametric Mann–Whitney U tests.

Anxiety and mood were assessed before the experiment with the STAI (State Trait Anxiety Inventory for State and Trait) and the PANAS (Positive and Negative Affective Schedule). Family conflict resolutions were assessed using a German questionnaire “Konfliktlösungsstile im Elternhaus” (KLSE). Social anxiety was measured with the SIAS (Social Interaction Anxiety Scale) and the SPS (Social Phobia Scale). Visual attention and concentration were assessed using the d2 (Aufmerksamkeits- und Belastungstest d2) and working memory performance was assessed using the German version of the Letter-Number-Span Test (“Buchstaben-Zahlen-Test”; BZT). Abbreviations: OXT, oxytocin; PLC, placebo.

**Figure 1:**
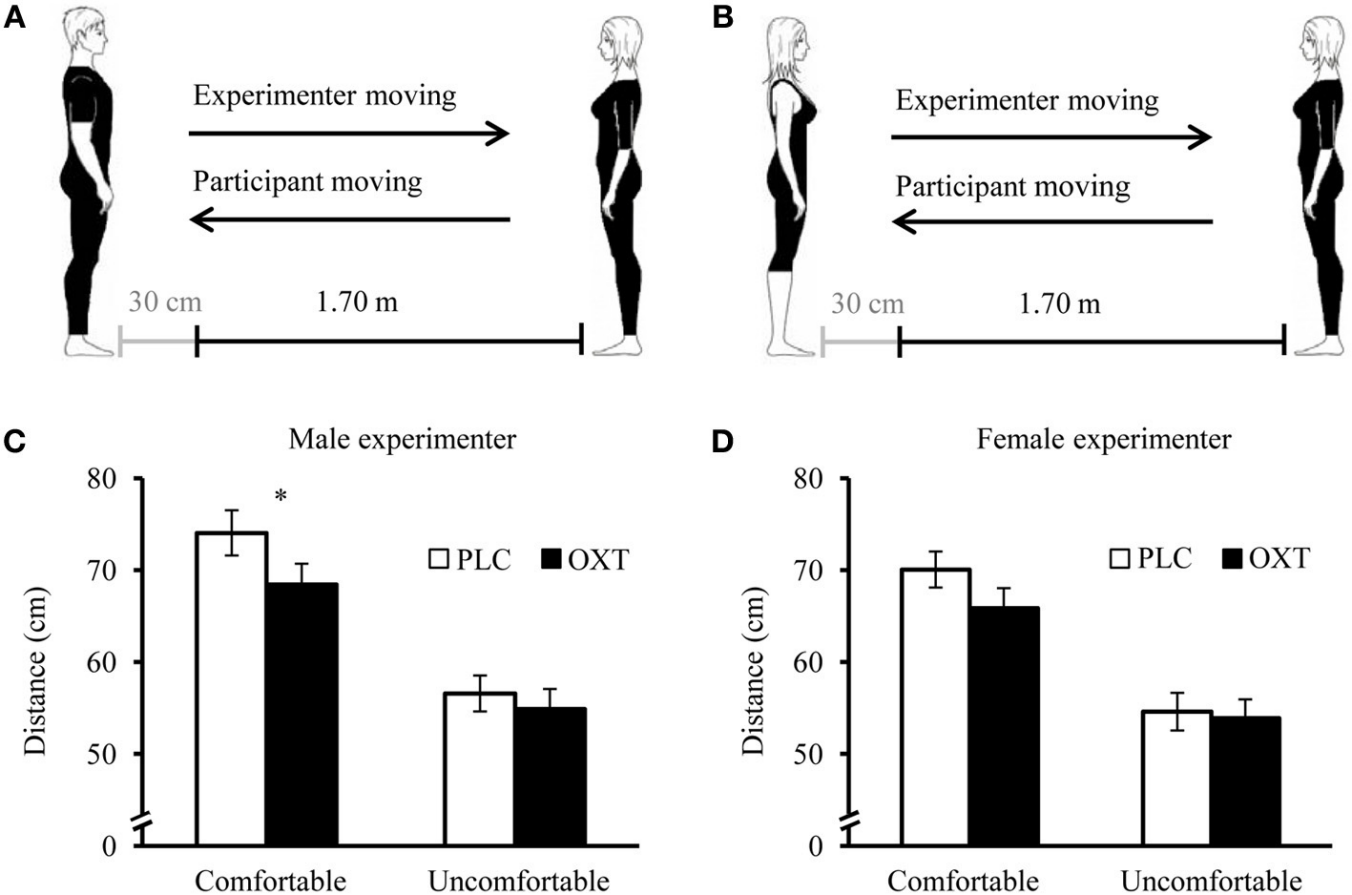
**Effects of OXT in the stop-distance-paradigm. Experimental setup for the male (A) and female experimenter (B)**. In the first half of the trials the experimenter was the one moving either toward (“far,” i.e., start distance of 2 m) or away from the subject (“close,” i.e., start distance of 30 cm), whereas in the second half the female volunteer was the one approaching or withdrawing. An additional condition was gaze direction, with the experimenter avoiding eye contact in half of the trials. Mean ideal (comfortable) distances and slightly uncomfortable distances across all conditions for the male **(C)** and female **(D)** experimenter. OXT significantly decreased the ideal distance that women maintained in relation to the unknown attractive man. Error bars indicate the standard error of the mean (s.e.m.). Abbreviations: OXT, oxytocin; PLC, placebo; ^*^P < 0.05.

In Experiment 1, we used the stop-distance paradigm to determine both the ideal distance for an interaction with an unfamiliar attractive male and an attractive female experimenter and the distance at which the subjects felt slightly uncomfortable (Figures 1A,B). Next, the subjects were asked to rate the attractiveness, sympathy and trustworthiness of the experimenters on a scale from 1 to 9 as well as their feelings during the testing (stress, embarrassment and reflection on their own emotions). Likewise, the experimenters evaluated the subjects on these scales. In Experiment 2, subjects performed an AA-task at which the speed of manual approach and avoidance (AA) response to stimuli varying in both valence and social content was measured. Subjects had to discriminate between positive (e.g., attractive men or beautiful landscapes) and negative (e.g., mutilations or dirt) pictures selected from the International Affective Picture System (IAPS) (Lang et al., 2005) as fast as possible by pulling a joystick toward (positive) or away (negative) from their own body. Since pleasant pictures in our previous study mostly displayed nude women, we had to replace these stimuli in the present study. Importantly, based on the normative valence and arousal IAPS ratings, the picture sets for female and male participants were comparable. Finally, Experiment 3 served as a non-social control task in which participants had to bisect a line in the middle as accurately as possible.

### Stop-distance paradigm

An adapted version of the stop-distance paradigm was used (Kennedy et al., 2009). Subjects met both experimenters for the first time. A standardized appearance of the experimenters was ensured for all subjects across all sessions and all subjects were tested in the same room. One half of the subjects started with the female experimenter and the other half started with the male experimenter. Subjects were positioned at one end of the room with their toes placed on a marking line. Trials were administered in a fixed order and each subject completed a practice trial before the start of the experiment. In the first half of the trials, the male or female experimenter moved at a natural gait either toward (“far,” i.e., start distance of 2 m) or away (“close,” i.e., start distance of 30 cm) from the subject, whereas in the second half, the female volunteer was the one moving. The experimenter avoided eye contact (EC) in half the trials, although subjects were not informed of this during initial task instruction. Subjects were asked to tell the experimenter to stop at their preferred distance in the first half of the trials and choose their ideal distance in the second half, when personally moving. The preferred or ideal distance was described as the distance at which the participants would feel most comfortable to have a conversation with a complete stranger. Subjects could fine-tune the distance by moving the experimenter slightly further backward or forward. After measuring the ideal distance, the experimenter/subject returned to the starting position and the same procedure was used to determine the slightly uncomfortable distance. All different trial conditions were repeated twice and in total there were 32 trials involving each experimenter. The final chin-to-chin distance was measured with a digital laser measurer (model DLR165K; Bosch; error = 0.003 m). Finally, the subjects were asked to estimate the distance that an average person would regard as optimal and as slightly uncomfortable.

### Approach-avoidance task

The pictures presented during the task were carefully adjusted for luminance using a self-written script in Matlab 7 (MathWorks). Each trial started with the presentation of a fixation cross for 1 to 2 s. Pictures were then presented randomly in four blocks (one block contained five pictures of each positive social/non-social and negative social/non-social category). Each picture was presented for 2000ms. Participants were instructed to place their head on a chin rest at a viewing distance of 50 cm to the computer screen. AA behavior was simulated by increasing or decreasing the picture size. Pulling the joystick replaced the picture by the same one enlarged by a factor of 1.1, while pushing the joystick reduced the picture size by a factor of 0.9. Reaction times were obtained by using the joystick displacement measurements for each trial. Trials showing an extreme reaction time (<200 or >1500 ms) or movements in the wrong direction were excluded. Participants (*n* = 9) who deviated more than two SDs from the mean reaction time were also excluded.

### Line bisection task

When participants have to bisect horizontally-oriented lines, they generally show a small left bias in near space (pseudo-neglect). At greater distances this bias shifts rightward and the rate at which this occurs can be taken as an index of the extent/size of the near space (Varnava et al., 2002). In the present paper, the line bisection task included three different distances (1, 2, and 3 m) from which participants had to bisect a horizontal line which was presented on a large screen (93 × 51 cm). From each distance the participants had to bisect the line 10 times by moving a cursor using a wireless mouse that was placed on a table next to the participants' right hand. We measured the bisection bias (in %) as dependent variable in this task, with positive and negative values indicating a rightward or leftward bias, respectively. The cursor had two different starting positions; it started at random either in the upper left or right corner of the screen. All trials for each distance were averaged in order to eliminate a possible effect of cursor starting position.

### Statistical analysis

Demographical, neuropsychological, and behavioral data were analyzed using SPSS 20 (SPSS Inc., Chicago, IL, USA). Quantitative behavioral data were compared by mixed-model analysis of variance (ANOVA) and Pearson's product-moment correlation was used for correlation analysis. Eta-squared and Cohen's d were calculated as measures of effect size. The assumption of sphericity was assessed with Mauchly's test, and for significant violations Greenhouse-Geisser's correction was applied. The assumption of normality was assessed with the Kolmogorov-Smirnov Test and in cases of a significant deviation non-parametric Mann–Whitney U-tests were used for group comparisons. For qualitative variables Pearson's chi-squared tests were used. All reported *P*-values are two-tailed, if not otherwise noted, and *P*-values of *P* < 0.05 were considered significant.

## Results

### Experiment 1: stop-distance paradigm

A repeated-measures ANOVA was carried out with the ideal or slightly uncomfortable distances as dependent variables, “eye contact” (EC; with or without), “person moving” (PM; experimenter or subject), and “starting position” (SP; close or far) as within-subject factors, and “treatment” (OXT or PLC) as between-subject factor. For the ideal distance involving the male experimenter as a stimulus, we observed main effects for EC [*F*_(1, 74)_ = 6.99, *P* = 0.01, η^2^ = 0.09], PM [*F*_(1,74)_ = 24.10, *P* < 0.01, η^2^ = 0.25], and SP [*F*_(1,74)_ = 15.80, *P* < 0.01, η^2^ = 0.18], and an interaction between PM and SP [*F*_(1,74)_ = 37.47, *P* < 0.01, η^2^ = 0.34]. The female participants maintained a larger distance between themselves and the experimenter if the latter avoided EC, and the interaction was qualified by a larger distance if the experimenter instead of the subject was moving in trials with a far SP. In the case of a close SP this effect was reversed. Importantly, there was a main effect of treatment with the OXT administration resulting in a closer approach across all conditions [*F*_(1,74)_ = 4.39, *P* = 0.04, η^2^ = 0.06, Figure 1C]. For the slightly uncomfortable distance, the main effects of PM [*F*_(1,74)_ = 16.43, *P* < 0.01, η^2^ = 0.18] and SP [*F*_(1,74)_ = 100.77, *P* < 0.01, η^2^ = 0.58] and the interaction between both factors [*F*_(1,74)_ = 11.68, *P* < 0.01, η^2^ = 0.14] remained significant, but there were no further significant results (all *P*s > 0.05).

For the ideal distance between the subjects and the female experimenter, we also found main effects of EC [*F*_(1,74)_ = 71.92, *P* < 0.01, η^2^ = 0.49], PM [*F*_(1, 74)_ = 4.97, *P* < 0.03, η^2^ = 0.06], and SP [*F*_(1, 74)_ = 21.79, *P* < 0.01, η^2^ = 0.23; Figure 1D]. There was no significant treatment effect (*P* = 0.14); for an additional analysis of a subsample of participants who rated the female experimenter as highly attractive and who exhibited the same OXT effect as with the male experimenter see the Supplementary Information. The ANOVA further revealed interactions between EC and PM [*F*_(1, 74)_ = 9.61, *P* < 0.01, η^2^ = 0.12], EC and SP [*F*_(1, 74)_ = 10.82, *P* < 0.01, η^2^ = 0.13] as well as SP and PM [*F*_(1, 74)_ = 29.26, *P* < 0.01, η^2^ = 0.28]. Participants showed a closer approach if the experimenter kept EC, if they were the person moving and if the SP was far. The EC effect was more pronounced if the starting position was close and if the experimenter was moving and a close SP led to a larger distance only if the subject was moving. For the estimate of the slightly uncomfortable distance between participants and the female experimenter, the main effects of PM [*F*_(1, 74)_ = 3.94, *P* = 0.05, η^2^ = 0.05] and SP [*F*_(1, 74)_ = 45.63, *P* < 0.01, η^2^ = 3.81] remained significant, but no further effects were detected (*P* > 0.05). The distances for all conditions are shown in Table 2.

**Table 2:** **Ideal and slightly uncomfortable distances (cm) in Experiment 1**.

	**OXT male experimenter (*N* = 38)**	**PLC male experimenter (*N* = 38)**	**OXT female experimenter (*N* = 38)**	**PLC female experimenter (*N* = 38)**
**COMFORTABLE DISTANCE**				
**Experimenter moves**				
From far (EC)	72.54 (18.75)	81.32 (18.71)	67.95 (15.85)	73.71 (14.33)
From far (NEC)	73.10 (18.03)	81.85 (17.08)	70.21 (14.43)	74.99 (13.79)
From close (EC)	73.38 (12.72)	76.69 (15.66)	69.11 (11.20)	70.76 (10.31)
From close (NEC)	73.15 (12.31)	79.93 (12.98)	73.08 (13.05)	75.42 (11.34)
**Participant moves**				
From far (EC)	64.35 (16.96)	71.26 (16.19)	62.48 (17.64)	69.05 (14.17)
From far (NEC)	64.42 (15.87)	72.34 (15.91)	64.30 (17.05)	69.33 (14.23)
From close (EC)	73.25 (15.05)	79.36 (17.95)	70.32 (15.11)	74.66 (16.60)
From close (NEC)	74.01 (16.12)	81.45 (16.85)	71.57 (15.61)	77.39 (18.50)
**UNCOMFORTABLE DISTANCE**				
**Experimenter moves**				
From far (EC)	51.21 (16.63)	55.73 (19.11)	49.45 (12.15)	52.65 (11.97)
From far (NEC)	50.88 (16.51)	55.41 (18.10)	50.36 (11.78)	51.71 (12.12)
From close (EC)	64.61 (12.77)	65.78 (13.51)	57.46 (9.68)	57.63 (11.52)
From close (NEC)	63.43 (12.87)	64.73 (13.16)	58.58 (10.07)	57.82 (11.72)
**Participant moves**				
From far (EC)	46.19 (12.83)	48.38 (12.55)	48.00 (13.18)	49.78 (12.80)
From far (NEC)	46.18 (13.12)	48.06 (11.81)	46.36 (11.92)	49.43 (11.36)
From close (EC)	61.73 (14.04)	65.01 (15.04)	56.99 (12.92)	57.98 (18.94)
From close (NEC)	62.71 (13.78)	65.78 (15.81)	56.39 (12.17)	59.79 (19.66)

Mean values (SD) are given. Abbreviations: EC, eye contact; NEC, no eye contact; OXT, oxytocin; PLC, placebo.

The OXT effect cannot be attributed to a generally altered space perception since no significant effect was observed for participants' judgment of the ideal and uncomfortable distances of an average woman (all *P*s > 0.05). Furthermore, the male experimenter perceived the OXT-treated female participants as more attractive [*F*_(1,71)_ = 11.23, *P* < 0.01, η^2^ = 0.14; Table 3] and likable [*F*_(1, 71)_ = 4.23, *P* = 0.04, η^2^ = 0.06] than PLC-treated subjects. There were also trends such that the experimenter reported increased reflective thinking about the women in the OXT group [*F*_(1, 71)_ = 3.34, *P* = 0.07, η^2^ = 0.05] and he rated women under OXT as more trustworthy [*F*_(1, 71)_ = 3.70, *P* = 0.06, η^2^ = 0.05]. None of these effects were evident for the female experimenter and the participants from the two treatment groups did not rate either experimenter significantly differently on any of these measures (all *P*s > 0.05). For the male experimenter, subjects' estimates in the OXT group regarding the ideal and slightly uncomfortable distances were negatively correlated with attractiveness (ideal: *r* = −0.33, *P* = 0.05, cf. Supplementary Figure 1; uncomfortable: *r* = −0.44, *P* < 0.01), likability (ideal: *r* = −0.35, *P* = 0.04; uncomfortable: *r* = −0.35, *P* = 0.04) and trustworthiness ratings (ideal: *r* = −0.30, *P* = 0.08; uncomfortable: *r* = −0.35, *P* = 0.04). No significant correlations were found in the PLC group (all *P*s > 0.05). For the female experimenter, the pattern of correlations was reversed, with significant associations being present only in the PLC group. The ideal and slightly uncomfortable distances were negatively correlated with likability (ideal: *r* = −0.55, *P* < 0.01; uncomfortable: *r* = −0.45, *P* < 0.01) and trustworthiness ratings (ideal: *r* = −0.59, *P* < 0.01; uncomfortable: *r* = −0.51, *P* < 0.01) and positively linked to embarrassment (ideal: *r* = 0.52, *P* < 0.01; uncomfortable: *r* = 0.64, *P* < 0.01) and stress (ideal: *r* = 0.37, *P* = 0.02; uncomfortable: *r* = 0.35, *P* = 0.03).

**Table 3:** **State measurements in Experiment 1**.

	**OXT (*N* = 38)**	**PLC (*N* = 38)**
**FROM MALE EXPERIMENTER**		
Stressful	2.06 (1.26)	1.87 (1.19)
Embarrassing	1.69 (0.83)	1.79 (0.96)
RooF	2.91 (2.09)	3.89 (2.46)
Likable	**7.37 (0.97)**	**6.68 (1.74)**
Attractive	**6.29 (1.15)**	**5.13 (1.71)**
Trustworthy	6.91 (1.36)	6.21 (1.73)
**FOR MALE EXPERIMENTER**		
Stressful	4.37 (2.05)	4.55 (2.31)
Embarrassing	2.58 (1.75)	3.16 (2.02)
RooF	5.34 (2.46)	4.97 (2.48)
Likable	7.45 (1.41)	7.71 (1.31)
Attractive	7.29 (0.98)	7.45 (0.95)
Trustworthy	7.47 (1.11)	7.37 (1.32)
**FROM FEMALE EXPERIMENTER**		
Stressful	3.46 (1.61)	2.95 (1.29)
Embarrassing	1.11 (0.31)	1.13 (0.41)
RooF	4.43 (1.44)	4.42 (1.15)
Likable	7.38 (0.92)	7.18 (1.09)
Attractive	6.86 (0.89)	6.53 (1.01)
Trustworthy	7.11 (1.10)	7.08 (1.22)
**FOR FEMALE EXPERIMENTER**		
Stressful	3.92 (1.88)	3.92 (2.16)
Embarrassing	2.24 (1.60)	2.58 (1.88)
RooF	4.71 (2.55)	4.89 (2.54)
Likable	8.26 (0.79)	8.08 (1.36)
Attractive	6.87 (1.19)	6.92 (1.32)
Trustworthy	8.39 (0.72)	8.08 (0.75)

Mean values (SD) are given. Bold numbers indicate a significant treatment difference (P < 0.05). Ratings were given on a scale from 1 (not true at all) to 9 (absolutely true). Abbreviations: OXT, oxytocin; PLC, placebo; RooF, Reflection on own feelings.

A comparison of the ideal and uncomfortable social distances obtained for heterosexual dyads in the present study with our previous findings (Scheele et al., 2012) in male participants revealed a significant main effect of the participants' gender [ideal distance: *F*_(1, 131)_ = 32.63, *P* < 0.01, η^2^ = 0.20; uncomfortable distance: *F*_(1, 131)_ = 105.01, *P* < 0.01, η^2^ = 0.45, cf. Supplementary Figure 2] and a significant interaction between the participants' gender and treatment for the ideal distance [*F*_(1, 129)_ = 11.69, *P* < 0.01, η^2^ = 0.08]. Under PLC, women kept a larger distance to the attractive opposite-sex experimenter than men [ideal distance: women PLC, 78 cm; men PLC, 56.73 cm; *t*_(65)_ = 6.64, *P* < 0.01, *d* = 1.66; uncomfortable distance: women PLC, 58.55 cm; men PLC, 36.77 cm; *t*_(65)_ = 8.75, *d* = 2.19]. In line with previous findings on empathy (Hurlemann et al., 2010), OXT reduced this a-priori sex difference and motivated women and men to approximate their preference for the social distance [ideal distance: women OXT, 71.03 cm; men OXT, 65.38 cm; *t*_(64)_ = 1.73, *P* = 0.09, *d* = 0.44; uncomfortable distance: women OXT, 55.87 cm; men OXT, 40.77, *t*_(64)_ = 5.85, *P* < 0.01, *d* = 1.48]. Considering effect sizes, the OXT effects were larger in men (ideal: η^2^ = 0.14, uncomfortable η^2^ = 0.08) than in women (ideal: η^2^ = 0.06, uncomfortable η^2^ = 0.01). This difference is likely related to a floor effect since a further reduction of the social space in women would lead to uncomfortable distances.

### Experiment 2: approach-avoidance task

**Figure 2:**
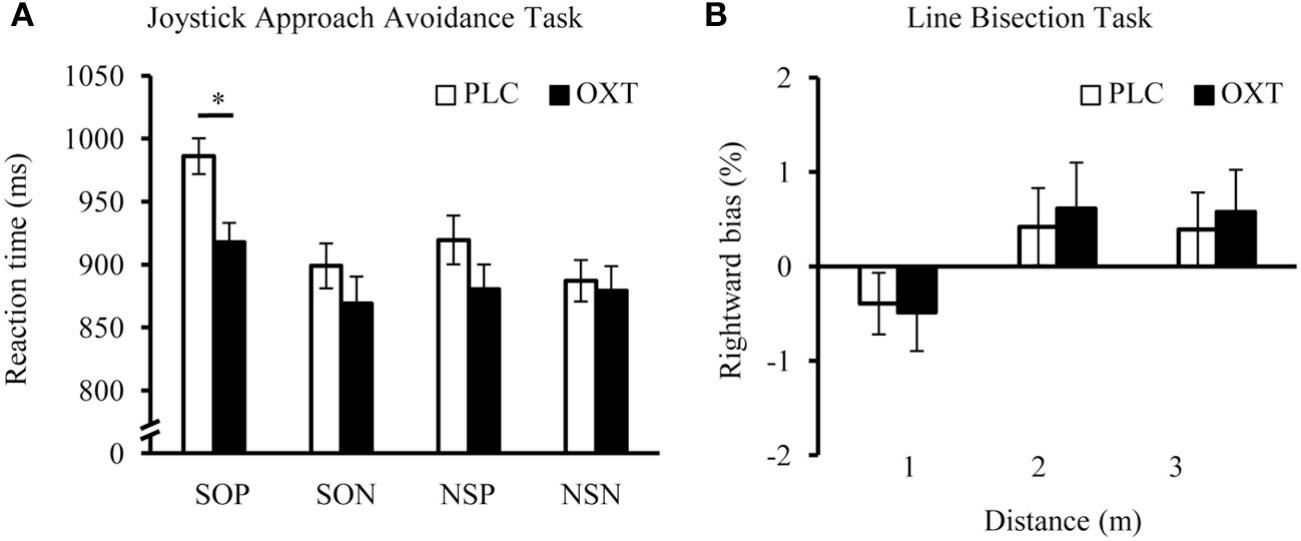
**Effects of OXT in the Joystick Approach-Avoidance (A) and Line Bisection Task (B)**. OXT specifically accelerated the approach toward social positive stimuli, but did not influence motor responses to negative or non-social cues. In the Line Bisection Task a right pseudo-neglect occurred if the line was presented in a near distance and this bias shifted leftward at farther distances. This shift reflects the extent of the peri-personal space and OXT had no effect on this non-social component. Error bars indicate the standard error of the mean (s.e.m.). Abbreviations: NSN, non-social negative; NSP, non-social positive; OXT, oxytocin; PLC, placebo; SON, social negative; SOP, social positive; ^*^P < 0.05.

A repeated-measures ANOVA with “sociality” (social or non-social) and “valence” (positive or negative) as within-subject factors, “treatment” (OXT vs. PLC) as between-subject factor and the reaction time as dependent variable yielded main effects of sociality [*F*_(1, 65)_ = 13.08, *P* < 0.01, η^2^ = 0.17] and valence [*F*_(1, 65)_ = 13.02, *P* < 0.01, η^2^ = 0.17], a trend for a treatment effect [*F*_(1, 65)_ = 3.72, *P* = 0.075, η^2^ = 0.05] and an interaction of sociality and valence [*F*_(1, 65)_ = 9.88, *P* < 0.01, η^2^ = 0.13]. All participants showed faster responses to non-social and to negative stimuli. The reaction time difference between positive and negative items was more pronounced for the social condition. Importantly, exploratory *post-hoc t*-tests revealed that OXT elicited faster approach behavior only for positive social stimuli [*t*_(65)_ = 2.45, *P* = 0.02, *d* = 0.61, Figure 2A] and had no effect on other categories (all *P*s > 0.05). In Supplementary Figure 3, the reaction times of the female participants in the present study are compared with the data obtained for male participants in our previous study (Scheele et al., 2012).

### Experiment 3: line bisection task

A repeated measures ANOVA with “distance” as a within-subject variable and “treatment” as a between-subject variable revealed a main effect of distance [*F*_(2,140)_ = 9.15, *P* < 0.01, η^2^ = 0.12; Figure 2B], but no further main or interaction effects (all *P*s > 0.05). In line with previous literature (Varnava et al., 2002), all participants displayed a right pseudo-neglect in near space and increasing left pseudo-neglect in far space. No significant differences were found between the two treatment groups in any of the three distances in the line bisection task. The pattern of results in all three experiments did not change if relationship status was incorporated as an additional between-subject factor and there were no interactions between treatment and the relationship status (all *P*s > 0.05).

## Discussion

In the present study, we aimed at elucidating the influence of OXT on AA behavior in healthy women. OXT decreased the social distance that female participants kept between themselves and an unfamiliar attractive and friendly male experimenter in Experiment 1 and accelerated the motor responses toward pleasant social scenes in Experiment 2. Notably, in Experiment 3 OXT did not alter lateral attentional bias used as an index of space perception and the extent/size of peri-personal space. Taken together, our results point to an evolutionarily adaptive mechanism by which OXT is promoting approach behavior in women specifically in positive social contexts.

Our findings are consistent with the biobehavioral synchrony model which posits that the temporal concordance of micro-level social behaviors in the gaze, vocal, affective, and touch modalities interacts with OXT responses to create dyad-specific affiliations (Feldman, 2012). In the present study, OXT may have influenced micro-level behaviors in the female participants such that they were perceived as more attractive and more likeable by the male experimenter. The negative association between these ratings and the social distance measured under OXT underscores that this improved interaction quality may have contributed to the closer approach. However, given the correlational nature of this finding, it is also conceivable that the enhanced approach behavior could itself have led to an altered perception of female subjects resulting in them being given higher ratings. If the OXT effect on social distance is mediated by subtle behavioral changes, this could explain the absence of a significant OXT effect in trials with a female experimenter where heterosexual women might be less perceptive or responsive to these subtle female behavioral cues. On the other hand, floor effects in the female-female interaction and uncomfortable distance conditions could have prevented a significant effect since in both conditions a smaller than the ideal distance determined in the mixed-sexes dyads was exhibited. The OXT effect in Experiment 1 was not influenced by the eye contact manipulation of the experimenters, but OXT could still have altered the participants' eye gaze. Furthermore, OXT may affect social interactions by enhancing pupil dilation in response to social stimuli (Leknes et al., 2013; Prehn et al., 2013) or by modulating the speed of head movements (Weisman et al., 2013).

In contrast to our previous study with male subjects (Scheele et al., 2012), we did not observe an interaction between treatment and relationship status in women. An evolutionary explanation for these sexually-dimorphic results arises from the different mating strategies that men and women may have developed to maximize their evolutionary fitness in view of their asymmetric parental investments (Buss et al., 1992). Men benefit from impregnating as many women as possible, while women benefit from choosing their mates more carefully (Levy and Kelly, 2010). These different mating strategies may have contributed to sex-specific jealousy behaviors, with men perceiving sexual infidelity as more threatening and women being more sensitive to emotional infidelity (Buss et al., 1992). Thus, men may endorse social approach and physical proximity as more distressing signals of potential infidelity compared to women. By increasing the reward value of their female partner (Scheele et al., 2013), OXT may further enhance these signals in men and trigger the avoidance of unfamiliar attractive females. For OXT effects on AA behavior in women, the relationship status appears to be less relevant. We acknowledge that mate choice preferences not only vary across the menstrual cycle but may also depend on the use of hormone-based contraceptives (Alvergne and Lummaa, 2010). We exclusively recruited women who took oral contraceptives since plasma OXT levels have been found to fluctuate throughout the menstrual cycle in normally cycling women, but not in women using oral contraceptive pills (Salonia et al., 2005). Women taking oral contraceptives display a significantly reduced sensitivity to social odors (Renfro and Hoffmann, 2013) and it has been proposed that OXT is responsible for the modulatory influence of romantic love on the ability of women to identify body odors of potential partners (Lundstrom and Jones-Gotman, 2009). Notwithstanding, by focusing on a homogenous sample we can exclude that any menstrual cycle-dependent hormonal changes contributed to our results.

Current perspectives on the central effects of OXT emphasize its pleiotropic contributions to sociality, although a substantial diversity in behavioral functions is evident across taxa (Anacker and Beery, 2013; Goodson, 2013). Our present results also highlight the crucial role of a social component for OXT actions in women, since in Experiment 2 facilatory OXT effects were restricted to positive social stimuli and we did not observe alterations in “non-social” peri-personal space in Experiment 3. Notably, these results are consistent with previous studies in men demonstrating that OXT specifically potentiated the social reinforcement advantage in a feedback-guided item-category association task (Hurlemann et al., 2010) and that it also affected social, but not financial information, in a decision-making paradigm (Evans et al., 2010). In both sexes, OXT improved the sensitivity to detect biological, but not non-biological motion (Keri and Benedek, 2009). The peptide also enhanced the suppression of electrophysiological oscillations in the mu/alpha and beta bands during the perception of biological motion, suggesting that OXT may act by allocating cortical resources to the social task (Perry et al., 2010). Clearly, it will be relevant to disambiguate whether these differential effects are due to a-priori higher salience of social stimuli or whether OXT truly requires a social component.

While the importance of sociality appears to be generalized, valence-dependent effects may differ between sexes. In men, OXT can augment defensive responses if threatening scenes are presented (Striepens et al., 2012). Furthermore, Theodoridou et al. (2013) argued that OXT may fulfill a prophylactic function by accelerating motor responses to disgusted facial expressions which signify threat to perceivers. Interestingly, in the latter study, no significant interaction between treatment and sex was found, however, the experimental design did not control for the hormonal status of the women and the applied stimulus set mainly consisted of face photographs. Emotional face photographs are highly salient and usually have no contextual information whereas ambiguous emotional stimuli and emotional scenes do contain contextual information.

Our data suggest a sexual-dimorphic effect of OXT on AA behavior such that in men it elicits social approach with caution even if the context is experienced as aversive and stressful, whereas in women social approach behavior appears to be restricted to safe social situations. The absence of any valence-specific effects in a non-social context lends further support to the idea that OXT specifically affects affiliative behavior. The discrepancy between women and men could perhaps be related to sex differences in brain OXT receptor distribution as well as in endogenous OXT levels. So far there is only a single autoradiography mapping study on the brains of eight males and four females which failed to establish any correlation between OXT receptor distribution or density and sex (Loup et al., 1991). There is also no evidence for higher or lower OXT plasma concentrations in women than in men (Feldman, 2012; Zhong et al., 2012), but there is an ongoing controversial debate about the relationship of peripheral and central measurement (Churchland and Winkielman, 2012). Consequently, future studies are warranted to test this hypothesis of sex-difference by employing modern OXT receptor mapping techniques and by measuring cerebrospinal fluid concentration in larger samples.

In conclusion, we here provide the first evidence that OXT in women facilitates approach behavior not only in response to various pleasant scenes but also in a real-life setting with other people. Notably, several psychiatric disorders have been associated with dysfunctional AA behavior. In particular, patients with social anxiety may benefit from a pharmacological enhancement of their approach behavior (Roelofs et al., 2009). Our findings emphasize the necessity to generate gender-tailored treatments by identifying features for each patient that constitute a safe social context in which OXT is most effective.

### Conflict of interest statement

The authors declare that the research was conducted in the absence of any commercial or financial relationships that could be construed as a potential conflict of interest.
