# Supplementary material for: Oxytocin facilitates social approach behavior in women
Source: Front Behav Neurosci. 2014 May 27;8:191. doi: 10.3389/fnbeh.2014.00191 (PMC4034412; doi:10.3389/fnbeh.2014.00191)
Supplement: Supplementary file 1 [file DataSheet1.DOCX]

***Supplementary Material***

**Oxytocin facilitates social approach behavior in women**

**Katrin Preckel^1,2^, Dirk Scheele^1,2^, Keith M. Kendrick^3^, Wolfgang Maier^1,4^, and René Hurlemann^1,2,*^**

^1 Department of Psychiatry and 2 Department of Medical Psychology, University of Bonn, 53105 Bonn, Germany^

^3 Key Laboratory for Neuroinformation, School of Life Science & Technology, University of Electronic Science & Technology of China (UESTC), 610054 Chengdu, P.R. China^

^4 German Center for Neurodegenerative Diseases (DZNE), 53175 Bonn, Germany.^

*** Correspondence:** Professor René Hurlemann, MSc MD PhD, Department of Psychiatry and Department of Medical Psychology, University of Bonn, Sigmund-Freud-Straße 25, 53127 Bonn, Germany, [renehurlemann@icloud.com](mailto:renehurlemann@icloud.com)

1. **Supplementary Data**

Given the complex design of experiment 1, we decided to stratify the analysis based on the only variable entailing a short temporal break between the trials. During this break, the male experimenter left the testing room and the female experimenter entered the room (or vice versa). Nevertheless, we also conducted a repeated-measures ANOVA with “gender” as an additional within-subject factor in order to control for a possible gender x treatment interaction. We found a main effect of gender (*F*_(1,74)_ = 12.67, *P* < 0.01, ƞ^2^ = 0.15), with female participants preferring a larger distance to the male experimenter (74.52 cm) than to the female experimenter (67.95 cm). However, there was no gender x treatment interaction and the pattern of significant results did not differ from the results pattern obtained by the stratified repeated measures ANOVAs in the main manuscript.

In a further supplementary analysis of Experiment 1, we restricted the sample to subjects (placebo (PLC) *n* = 28, oxytocin (OXT) *n* = 27) who perceived the female experimenter as highly attractive (rating > 7). A univariate ANOVA with the ideal distance as dependent variable revealed a significant main effect of treatment (*F*_(1,53)_ = 4.58, *P* < 0.04, ƞ^2^ = 0.08). Applying the same selection criteria (rating > 7) to the attractiveness ratings for the male experimenter yielded a sub-sample of 62 participants (PLC *n* = 33, OXT *n* = 29). In this sub-sample the treatment effect remained significant (*F*_(1, 60)_ = 4.13, *P* < 0.05, ƞ^2^ = 0.06). In the small sample of 14 participants who did not perceive the male experimenter as attractive (i.e. attractiveness rating < 7), the OXT effect did not reach statistical significance (*F*_(1, 12)_ = 2.10, *P* = 0.17, ƞ^2^ = 0.15), but OXT-treated subjects (Mean = 76.50 cm, SD = 15.27cm) still preferred a smaller distance than PLC-treated participants (Mean = 92.25 cm, SD = 25.90cm).

1. **Supplementary Figures**

**
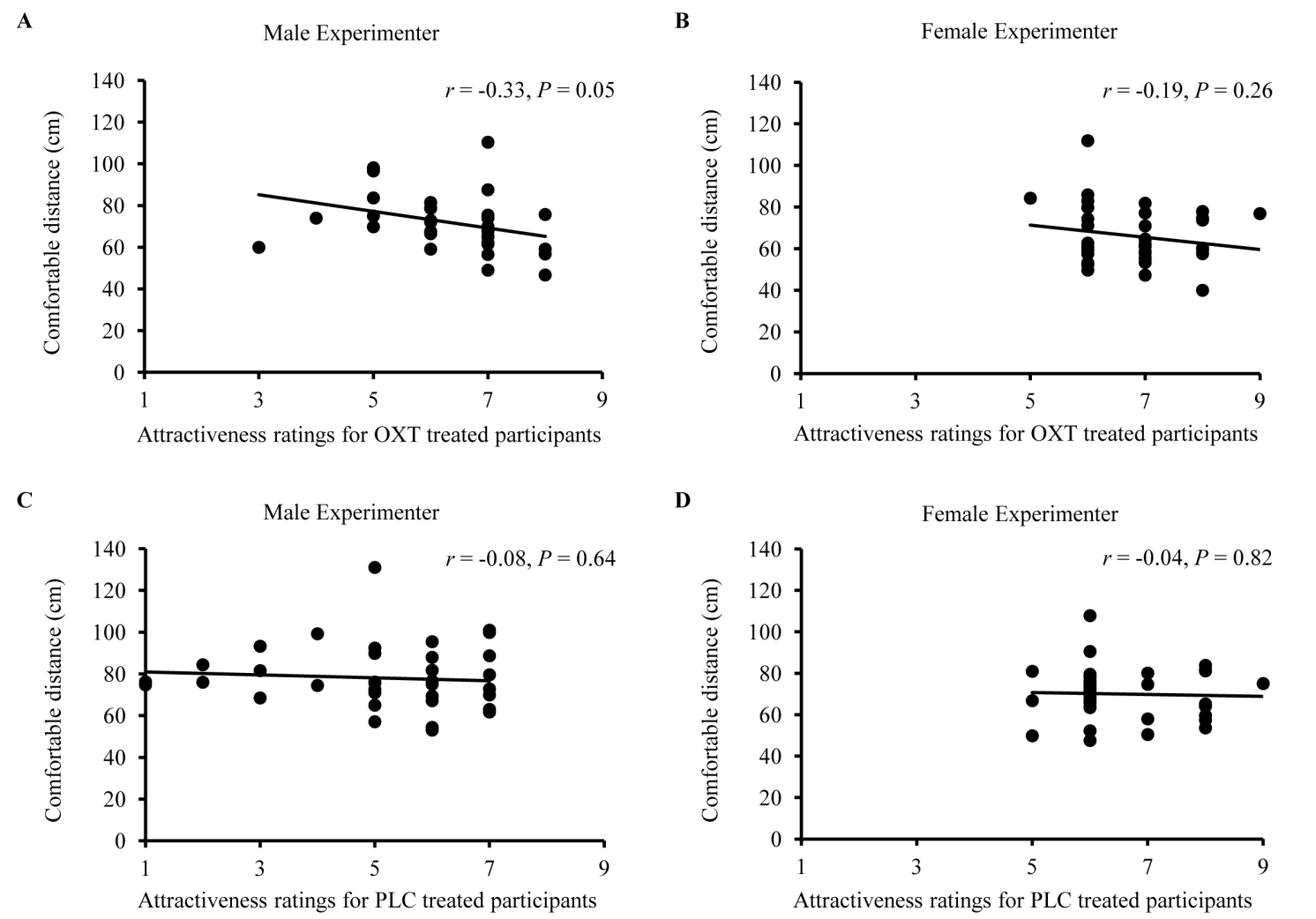
**

**Supplementary Figure 1**

Associations between the ideal social distance and the participants’ attractiveness as rated by the experimenters. A significant correlation was evident only for OXT-treated participants and the male experimenter (**A**), but not for the female experimenter (**B**). Under PLC, there was neither a correlation for the male (**C**) nor the female experimenter (**D**). Abbreviations: OXT, oxytocin; PLC, placebo.


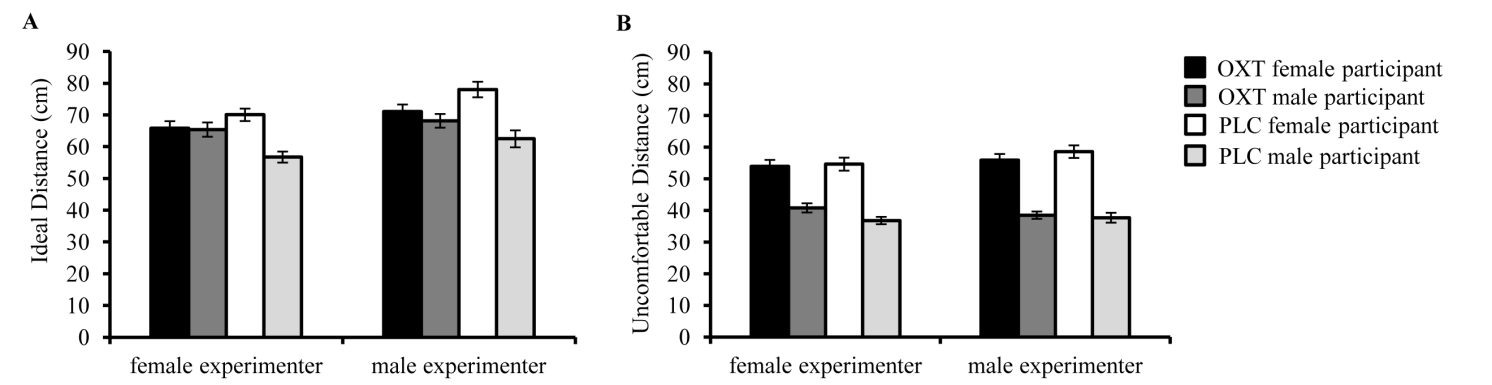


Supplementary Figure 2

The effect of OXT on the ideal (**A**) and uncomfortable (**B**) social distance between women and men. Overall, female participants preferred a larger distance than male participants and this difference became smaller if OXT was administered. The increase of social distance after OXT treatment in men was driven by pair-bonded participants (cf. Scheele et al., 2012). Data for male participants are derived from Scheele et al. (2012). Error bars indicate the standard error of the mean (SEM). Abbreviations: OXT, oxytocin; PLC, placebo.


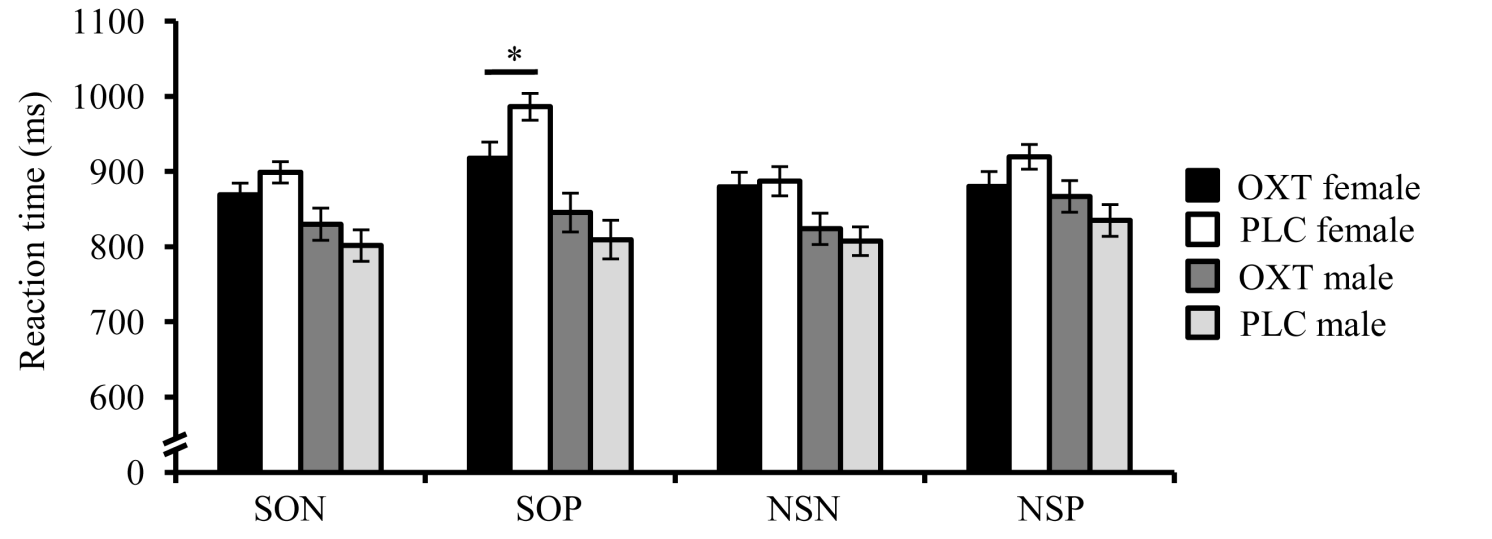


**Supplementary Figure 3**

The effect of OXT on reaction time based approach and avoidance behavior. OXT accelerated the approach of positive social stimuli in women. OXT had no general effect on approach and avoidance behavior in men, but selectively decelerated the approach of positive social stimuli in pair-bonded men (cf. Scheele et al., 2012). Data for male participants are derived from Scheele et al. (2012). Error bars indicate the standard error of the mean (SEM). Abbreviations: NSN, non-social negative; NSP, non-social positive; OXT, oxytocin; PLC, placebo; SON, social negative; SOP, social positive; * *P* < 0.05.

**Supplementary References**

Scheele, D., Striepens, N., Güntürkün, O., Deutschländer, S., Maier, W., Kendrick, K.M., and Hurlemann, R. (2012). Oxytocin Modulates Social Distance between Males and Females. *J Neurosci* 14**,** 16074-16079. doi: 10.1523/JNEUROSCI.2755-12.2012.
